# Association between the concentration of fine particles in the atmosphere and acute respiratory diseases in children

**DOI:** 10.1590/S1518-8787.2017051006523

**Published:** 2016-12-19

**Authors:** Antônio Paula Nascimento, Jane Meri Santos, José Geraldo Mill, Juliana Bottoni de Souza, Neyval Costa Reis, Valdério Anselmo Reisen

**Affiliations:** I Departamento de Tecnologia Industrial. Universidade Federal do Espírito Santo. Vitória, ES, Brasil; II Departamento de Engenharia Ambiental. Universidade Federal do Espírito Santo. Vitória, ES, Brasil; III Departamento de Ciências Fisiológicas. Universidade Federal do Espírito Santo. Vitória, ES, Brasil; IV Programa de Pós-Graduação em Saúde Coletiva. Universidade Federal do Espírito Santo. Vitória, ES, Brasil; V Departamento de Estatística. Universidade Federal do Espírito Santo. Vitória, ES, Brasil

**Keywords:** Child, Particulate Matter, Adverse Effects, Air Pollution, Respiratory Diseases, Epidemiology

## Abstract

**OBJECTIVE:**

To analyze the association between fine particulate matter concentration in the atmosphere and hospital care by acute respiratory diseases in children.

**METHODS:**

Ecological study, carried out in the region of Grande Vitória, Espírito Santo, in the winter (June 21 to September 21, 2013) and summer (December 21, 2013 to March 19, 2014). We assessed data of daily count for outpatient care and hospitalization by respiratory diseases (ICD-10) in children from zero to 12 years in three hospitals in the Region of Grande Vitória. For collecting fine particulate matter, we used portable samplers of particles installed in six locations in the studied region. The Generalized Additive Model with Poisson distribution, fitted for the effects of predictor covariates, was used to evaluate the relationship between respiratory outcomes and concentration of fine particulate matter.

**RESULTS:**

The increase of 4.2 µg/m^3^ (interquartile range) in the concentration of fine particulate matter increased in 3.8% and 5.6% the risk of medical care or hospitalization, respectively, on the same day and with six-day lag from the exposure.

**CONCLUSIONS:**

We identified positive association between outpatient care and hospitalizations of children under 12 years due to acute respiratory diseases and the concentration of fine particulate matter in the atmosphere.

## RESUMO

**OBJETIVO:**

Analisar a associação entre a concentração de material particulado fino na atmosfera e atendimento hospitalar por doenças respiratórias agudas em crianças.

**MÉTODOS:**

Estudo ecológico, realizado na Região da Grande Vitória, ES, no inverno (21 de junho a 21 de setembro de 2013) e no verão (21 de dezembro de 2013 a 19 de março de 2014). Foram avaliados dados de contagem diária de atendimentos ambulatoriais e hospitalizações por doenças respiratórias (CID-10) em crianças de zero a 12 anos em três hospitais da Região da Grande Vitoria. Para a coleta de material particulado fino foram utilizados amostradores portáteis de partículas instalados em seis locais na região estudada. O Modelo Aditivo Generalizado com distribuição de Poisson, ajustado para efeitos das covariáveis preditoras, foi utilizado para avaliar a relação entre os desfechos respiratórios e a concentração de material particulado fino.

**RESULTADOS:**

O incremento de 4,2 µg/m^3^ (intervalo interquartílico) na concentração de material particulado fino aumentou em 3,8% e 5,6% o risco de atendimento ou internação, respectivamente, no mesmo dia e com seis dias de defasagem da exposição.

**CONCLUSÕES:**

Foi identificada associação positiva entre atendimentos ambulatoriais e hospitalizações de crianças com até 12 anos devido a doenças respiratórias agudas e a concentração de material particulado fino na atmosfera.

## INTRODUCTION

Air pollution is associated with problems to human health and loss of quality of life^16^. Air pollutants fall into two categories: gases (O_3_, NO_2_, SO_2_, CO, for example) and particulate matter (PM), with different grain sizes and chemical composition. These pollutants have been linked to adverse health effects even in low concentrations^9^ ^,^ ^18^, especially PM, the most responsible for health problems related to the respiratory system^24^. The harmful effect to human health caused by PM depends both on its concentration in the inhaled air and on its granulometry and chemical composition. Chemical composition, granulometry, and concentration of PM in the atmosphere depend, especially, on its sources, which can be natural or anthropogenic (processing, mining, construction industries; vehicular emissions; among others), and on the emission intensity.

The PM is classified according to its aerodynamic diameter (varies from few nanometers to 100 µm)^3^. In addition to sedimentary particles (SP) that cause discomfort, the inhalable PM harmful to health are divided into three groups: particles with diameter lower than or equal to 10 µm (PM_10_), fine particles with diameter lower than or equal to 2.5 µm (PM_2.5_), and ultrafine particles with diameter lower than or equal to 0.1 µm^10^ ^,^ ^24^. The harmful effects of PM_2.5_ occur in the short term, by direct action on the airways, and in the long term, because, once inhaled, it can reach the alveoli, get to the bloodstream, and affect other organs besides the lungs^5^. Thus, PM_2.5_ presents potential health risk even when in relatively low concentrations in the atmosphere, i.e., even when its atmospheric concentration is below the maximum levels of tolerance established by the World Health Organization (WHO) and by the main environmental regulatory agencies of the world^15^ ^,^ ^24^.

Studies show the association between air pollution and incidence of respiratory, cardiovascular, neurological diseases and several types of cancer^10^ ^,^ ^14^ ^,^ ^24^. The association with respiratory diseases is stronger and direct, and the most vulnerable groups are children, older adults, and those with pre-existing respiratory diseases, especially asthma, chronic bronchitis, and chronic obstructive pulmonary disease^3^ ^,^ ^5^ ^,^ ^19^. Epidemiological studies quantify the impact of PM_2.5_ concentration and the incidence of acute morbid events in an attempt of seeking minimum levels of safety for exposure^13^ ^,^ ^15^. The triggering of asthma, bronchitis, and pneumonia attacks is often associated with the increase of air pollution by PM^3^ ^,^ ^17^ and gases.

However, the relationship between concentration of PM in the atmosphere and incidence of diseases presents inconsistencies, because concentration, by itself, may not be the only reason for harmful effects to health. The presence of some chemical element in PM, even in small concentrations, may also be associated with the occurrence of diseases^9^ ^,^ ^14^. There are few Brazilian studies relating PM_2.5_ with problems to human health^17^.

This study aimed to evaluate the association between concentration of PM_2.5_ in the atmosphere and incidence of acute respiratory events in children from zero to 12 years old.

## METHODS

**Figure 1:**
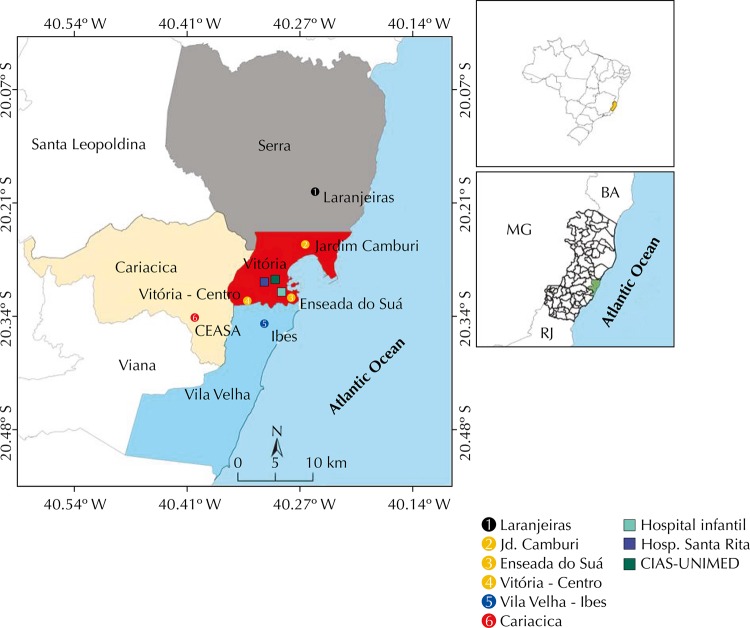
Location of sampling stations of fine particulate matter (1 to 6), and hospitals included in the study. Grande Vitória Region, ES, Southeastern Brazil, 2013-2014.

This is an ecological study, carried out in the Region of Grande Vitória (RGV), Espírito Santo, in the winter (June 21 to September 21, 2013) and summer (December 21, 2013 to March 19, 2014). Samples of PM_2.5_ in the atmosphere were collected in six stations located in the cities of Serra, Vitória, Vila Velha, and Cariacica (urban and industrialized region) in those periods (Figure 1). The samples were collected on alternate days during winter and summer, in continuous periods of 24 hours, with start of measurement on midnight. The concentration of PM_2.5_ in RGV was determined by the arithmetic mean of the values detected in the six stations.

We used Mini-Vol portable samplers (Airmetrics – USA), developed in conjunction with the United States Environmental Protection Agency (U.S.EPA)^a^ and already used in other studies^1^ ^,^ ^23^. The filters for sampling PM_2.5_, with 47 mm diameter and efficiency for retention of particles of up to 0.3 μm, met the standard MB-3422^b^ and recommendations of the code 40 CFR Part 50 Appendix L^c^. The filters were accommodated in desiccator for 24 hours (20°C to 23°C and humidity between 30.0% and 40.0%) for initial weighing and gravimetric quantification after neutralization of static electrical charges^a^. We used an analytical balance (Sartorius – Germany), with sensitivity of 1 μg, for the gravimetric analysis. The mean daily concentration of PM_2.5_ (μg/m^3^) was calculated by the ratio between the mass of PM_2.5_ and the total volume of sampled air in Mini-Vol, considering the mean uncertainties of weighing of the white filters of laboratory and field^a^ ^,^ ^b^.

Weather variables (temperature, relative humidity) were obtained from the Weather Network located at Eurico de Aguiar Salles Airport^d^.

The data of emergency care and hospitalization of children from zero to 12 years were obtained during the periods of measurement of PM_2.5_ in three hospitals: Hospital Infantil Nossa Senhora da Glória (HINSG), main pediatric emergency room in the region, Hospital Unimed Vitória, and Hospital Santa Rita de Cássia, the first being public and the other two, private. These hospitals do not represent the totality of care, but show a significant portion of the urgency/emergency care in RGV, mainly by the high volume of patient care at HINSG, specialized in pediatric patients. We considered as “acute respiratory events” those encoded from JJ00 to JJ99 in the International Classification of Diseases (ICD-10): diseases of the upper airways (rhinitis, sinusitis, and pharyngitis) and lower airways (asthma, pneumonia, bronchitis, bronchiolitis, and obstructive pulmonary diseases). We have included only the care to children with home accommodation in the four cities of RGV (Figure 1). The care of a patient already previously seen was considered as new event if the interval between the sessions was higher than seven days. For an event to be inserted in the database of this study, the medical record should present: name, home address of the child, sex, date of birth, and diagnosis.

The association between atmospheric concentration of PM_2.5_ and daily incidence of acute respiratory events was investigated by the Generalized Additive Model (GAM) with Poisson distribution. The use of GAM has been explored in other studies^6^ ^,^ ^7^ ^,^ ^11^. We included in the model, as explanatory covariates (besides the concentration of PM_2.5_), the collection period (winter/summer), seasonality of short duration (days of the week), holidays, seasonality of long duration (number of elapsed days), and weather variables (temperature and relative humidity)^2^ ^,^ ^8^. GAM enables the modeling of the non-linear relationship between explanatory and outcome variables, including parametric and non-parametric functions to enable the data curve fitting. The outcome variable (hospitalization/emergency care) corresponds to a discrete probability distribution because it is a process of counting non-negative integers, which follows Poisson distribution.

Be {Y_t_} , t = 1,..., N, a time series of count of non-negative integers. The conditional density function of {Y_t_} given passed information (*F*_t-1_), denoted by {Y_t_ | *F*_t-1_}, has Poisson distribution, with mean µ_t_, if it meets:


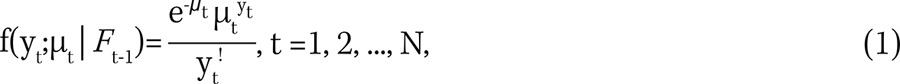


(1)

where y_t_ represents hospitalization/emergency care at moment (day) t.

Be X = (X_1_, X_2_, …, X_p_) a vector of p explanatory covariates. The curve that describes the relationship between y_t_ and the covariates X is obtained by the logarithmic transformation of µ_t_ given by equation (2).


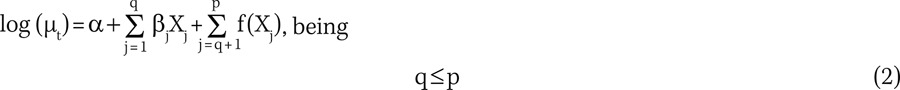


, being

q <= p (2)

where α is the model intercept; b_↓j_ (j=1,...,q) represent the linear regression coefficients associated with the concentration of PM_2.5_ and with indicative covariates for the days of the week, holidays, seasons (winter/summer); and f (X_j_) are smoothing spline functions for confusion variables (temperature and humidity) and temporal trend variables (number of elapsed days)^17^ ^,^ ^22^. The spline function allows the control of the nonlinear dependence between the covariates and outcome variable^8^ ^,^ ^17^.

The GAM fitting was obtained after analyses considering the temporal trend, seasonality, data correlation with time by the auto correlation function (ACF). We applied usual tests of residuals and goodness of fit using the Akaike criterion (AIC).

The relative risk (RR) is a measure of occurrence between the probability of an epidemiologic event^4^ occurring given the exposure to certain level of the exposure factor in relation to those affected by the same event and not exposed to the factor. In this study, RR refers to the increased risk of acute respiratory events occurring given the exposure of concentration levels of PM_2.5_.

For the GAM with usual Poisson distribution, RR is expressed by equation (3).


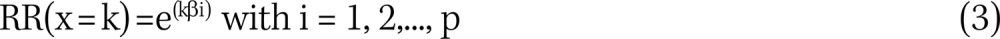


(3)

Being k the concentration variation of PM_2.5_, here considered by the difference between the third and first quartiles, and bˆ the coefficient estimated by GAM.

Data were supplied as mean and standard deviation (SD) and outcome as counts. We used a significance level of 5%. The process of modeling and statistical analysis were made on the R platform.

The project was approved by the Research Ethics Committee (CEP) of the Brazilian Ministry of Health (CAAE 080 197 12.6.0000.5542) and other committees of the hospitals.

## RESULTS

8,987 events were recorded by acute respiratory disease in children from zero to 12 years in both periods of collection; 64.0% of them occurred in the winter, with a daily mean of 58 (SD = 13). The mean of events was 36 (SD = 6) events/day in summer. The number of patient care was slightly higher in male children in both seasons. There was a predominance of care to children from zero to two years old, and approximately 95.0% of cases did not require hospitalization (Table 1).

**Table 1:** Descriptive statistics of acute respiratory events in children from zero to 12 years in three hospitals, concentration of fine particulate matter, and weather conditions in winter and summer. Grande Vitória Region, ES, Southeastern Brazil, 2013 to 2014.

Parameters	Winter 2013	Summer 2013/2014
Total events	5,786	3,201
Incidence (events/day)	58 (SD 13)	36 (SD 16)
Nature of the event	n (%)	n (%)
Outpatient care	5,446 (94.0)	3,053 (95.0)
Hospitalization	340 (6.0)	148 (5.0)
Sex		
Male	3,083 (53.0)	1,707 (53.0)
Female	2,703 (47.0)	1,494 (47.0)
Age group (years)		
0-2	3,127 (54.0)	2,010 (63.0)
3-6	1,490 (26.0)	891 (28.0)
7-12	1,169 (20.0)	300 (9.0)
PM_2.5_ (µg/m^3^)		
Mean (SD) (median)	11.4 (3.1) (11.2)	12.2 (3.8) (10.5)
1^st^ quartile	8.9	9.6
3^rd^ quartile	13.5	13.8
Minimum-Maximum	5.9-19.7	6.7-23.2
Temperature (^o^C)		
Mean (SD) (median)	23.1 (1.7) (22.9)	28.2 (1.1) (28.0)
1^st^ quartile	21.7	28.0
3^rd^ quartile	24.5	29.0
Minimum-Maximum	20.0-26.1	23.0-30.0
Relative humidity (%)		
Mean (SD) (median)	77.0 (6.4) (77.6)	71.3 (7.2) (69.0)
1^st^ quartile	73.5	66.0
3^rd^ quartile	81.4	74.0
Minimum-Maximum	61.0-92.3	62.0-93.0
Minimum-Maximum	1.0-358.0	2.0-360.0

Incidence given as mean; standard deviation (SD).

The mean concentration of PM_2.5_ for the whole period was 11.8 µg/m^3^ (SD = 3.5), with interquartile interval of 4.2 µg/m^3^ (Table 2).

**Table 2:** Estimated coefficients of the Generalized Additive Model, considering six-day lag between exposure to fine particulate matter and the hospital event. Grande Vitória Region, ES, Southeastern Brazil, 2013 to 2014.

	Estimated coefficients of the GAM				
	
	Estimate	Standard error	z value	p	
Intercept	2.149	0.557	3.857	0.000	***
Tuesday	-0.173	0.049	-3.500	0.000	***
Thursday	-0.197	0.049	-4.029	0.000	***
Saturday	-0.1227	0.048	-2.513	0.012	*
Holiday1	0.586	0.095	6.176	0.000	***
Winter	0.801	0.102	7.874	0.000	***
Temperature	0.049	0.013	3.645	0.000	***
PM_2.5_ *lag*3	0.013	0.006	2.106	0.035	*

Holiday1: Independence and foundation day of Vitória

Significance 0 ‘***’ 0.001 ‘**’ 0.01 ‘*’ 0.05 ‘.’ 0.1 ‘ ’ 1

The temperature and humidity values were within normal climatic means foreseen for winter and summer in RGV, with mean values of 22.3°C for temperature and 78.0% for relative humidity.

**Figure 2:**
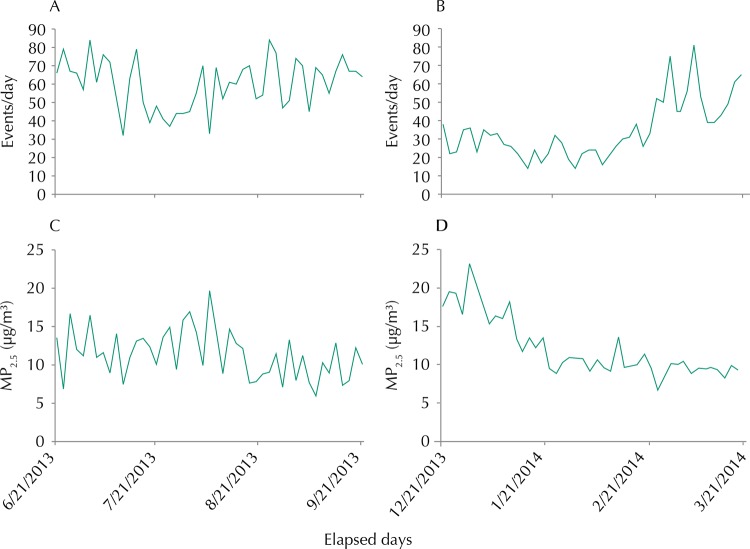
Time series of the number of acute respiratory events during (a) winter and (b) summer. Time series of concentration of fine particulate matter during (c) winter and (d) summer. Region of Grande Vitória, ES, Southeastern Brazil, 2013-2014.

**Figure 3:**
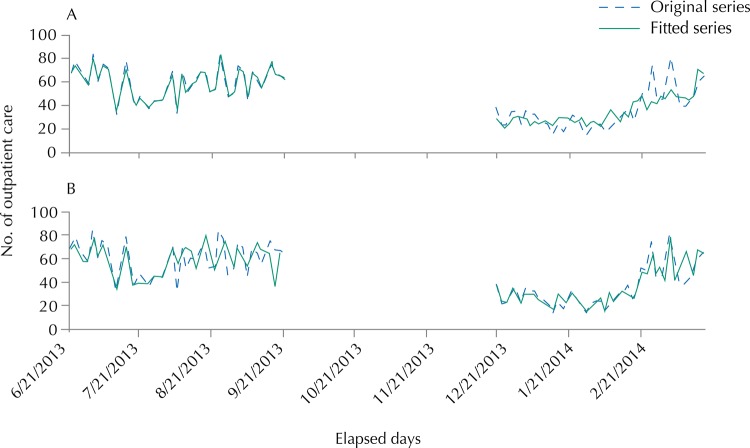
Fitting of the Generalized Additive Model considering exposure to pollutant (a) in the current day and (b) with six-day lag. The full line represents the original series and the dashed line represents the series fitted to *lag* 3. Region of Grande Vitória, ES, Southeastern Brazil, 2013 to 2014.

The events had higher and more uniform fluctuation in winter ((Figure 2, A). In summer ((Figure 2, B), we observed an increasing trend in the number of events and low daily fluctuation in the beginning of the period. The events showed a behavior similar to winter in the last month of summer. The mean concentration had high fluctuation, but was relatively uniform throughout the winter (Figure 2, C), while in summer (Figure 2, D) we observed a decreasing trend in the concentration of PM_2.5_ and low fluctuation in almost the entire period. In none of the days, the mean concentration exceeded the threshold of 25 µg/m^3^, maximum safety limit recommended by the WHO. There was agreement in the fluctuations of the original time series and of that modeled in winter, while the agreement was less evident in summer (Figure 3, A). Considering that some events included in this study, such as those of infectious and allergic nature, usually occur with a lag in time between the increased concentration of the pollutant and the observation of outcomes, the modeling was repeated with a lag of up to six days between the measure of PM_2.5_ and the occurrence of events. In this case, the agreement for the winter period was not as good as that considering the exposure to PM_2.5_ in the current day. However, the agreement has improved substantially for the summer (Figure 3, B). The best fit occurred when the tested temporal lag was of six days.

The results fitted in GAM for the six-day lag resulted in an estimate of the parameter (bˆ) of 0.0129 (SD = 0.006) (Table 2).

We estimated the RR of 1.0382 (95%CI 0.990–1.089) and of 1.056 (95%CI 1.003–1.111) for each increment of 4.2 µg/m^3^ in the concentration of PM_2.5_ in the RGV, respectively, for exposure at the current day and six-day lag between exposure and outcome. Therefore, children from zero to 12 years living in RGV suffered increase of almost 6.0% of probability to be suffering from acute episode of respiratory disease requiring hospital care at every increment of 4.25 µg/m^3^ of PM_2.5_ in the inhaled air.

## DISCUSSION

The results of this study showed RR of 1.02 (95%CI 0.99–1.09) to the increase of 4.25 µg/m^3^ in the concentration of PM_2.5_ for exposure at the current day, and RR of 1.06 (95%CI 1.00–1.11) to six-day lag from the exposure to PM_2.5_ of children under 12 years being affected by acute respiratory diseases, without significant differences between sexes. Previous studies investigated the effect of the lag between exposure to the pollutant and hospital event and indicated that, for larger lags, there is increased risk of association with short-term adverse effects of acute diseases^8^ ^,^ ^11^ ^,^ ^13^. Similar to other studies, the risk of disease in vulnerable groups was most evident with time lag between exposure and outcome^11^ ^,^ ^22^. Studies show that the PM in the atmosphere causes more significant effects on more vulnerable population subgroups, including children, especially in the early years of life, older adults, and those with preexisting diseases^3^ ^,^ ^10^ ^,^ ^19^.

The direct contact between particulate matter and respiratory system predisposes the occurrence of symptoms in short period of time. Children exhibit the body respiratory volume minute/weight greater than adults, which contributes to higher exposure to atmospheric pollutants^3^ ^,^ ^12^. Other factors, such as increased responsiveness of the epithelium and smooth muscle to PM_2.5_, can contribute to make this age group more susceptible to the effects of pollution, mainly children of up to two years, as observed in this study. However, the data collected is insufficient to detect different vulnerabilities between the age groups. Additional studies with longer series or data collection on a larger contingent of exposed people may explain this finding. For both modelings, in the current day or over six days of exposure, the children exposed presented RR of about 4.0% and 6.0%, respectively. There was an increase of at least 2.0% in the value of RR estimated by the lagged GAM, when compared to the GAM in the current day. Other authors^8^ ^,^ ^11^ ^,^ ^17^ observed increase in the number of hospitalizations by respiratory diseases related to the growth of particles smaller than 10 µg/m^3^.

Stafoggia et al.^22^ (2013) found a strong association in the short-term between the increase of 10 µg/m^3^ of PM_10_ and PM_2.5_ with hospitalizations by respiratory diseases in eight Mediterranean cities in Europe. They estimated variation in the risk of 1.15% (95%CI 0.21–2.11) for PM10 and of 1.36% (95%CI 0.23–2.49) for PM2.5 to exposure at the same day and five-day lag, respectively. Another study conducted in Copenhagen observed that, to an increase of 10 µg/m^3^ of PM2.5, the RR was 1.09 (95%CI 1.04–1.13) for outcomes by asthma in children and adolescents up to 18 years^13^. In Cordoba, Argentina, an increase of 10 µg/m^3^ of PM10 resulted in RR of 3.44% (95%CI 2.93–3.95) in male children younger than six years old and RR of 3.46% (95%CI 2.93–3.95) for female children in winter, spring, and autumn; while the RR was 3.21% (95%CI 2.85–3.88) for males and 3.46% (95%CI 2.87–3.90) for females in summer^2^.

In Piracicaba, SP, a research noted association between exposure to PM_2.5_ and hospitalization by respiratory diseases in children up to 10 years, with RR of 1.01 (95%CI 1.00–1.02) on *lag* 1 and of 1.01 (95%CI 1.00–1.02) on *lag* 3^7^. A larger emission of PM_2.5_ due to biomass burning was associated with higher prevalence of hospitalizations by respiratory diseases in children up to four years and older adults over 65 years in 186 cities of Mato Grosso during 2004^20^.

Previous studies in RGV showed association of hospital care by asthma in children up to six years and concentration of PM_10_ in the atmosphere^6^. Another study, also in RGV, presented an increase of 3.0% in the RR of hospital care by respiratory diseases in children younger than six years for the increase of 10.49 µg/m^3^ in the concentration of PM_10_ ^21^. These results together suggest that the finest PM seems to be more aggressive for the respiratory system of children, mainly for the younger ones.

We chose to consider children morbidity data from both public hospitals belonging to the Brazilian Unified Health System and private hospitals attended by the lower economic strata of the population. This aimed to exclude the economic strata as a variable in our analysis and their influence on exposure to risks of respiratory outcomes due to air pollution. However, we consider that the care carried out in these hospitals reflect the circadian fluctuations in all the population exposed to air pollution in RGV due to the PM2.5 emissions in the region.

The mean concentration of 24 hours of PM2.5 during the investigated period was 11.80 µg/m^3^. The mean concentration of PM2.5 was slightly higher in the summer, probably due to the rainfall that was below the expected mean for the period. In some periods RGV was under the influence of the South Atlantic Subtropical Ridge (SASR) in the summer. The winter of 2013 was rainier than usual, which may have contributed to the PM reduction in the atmosphere. In summer, precipitation was below the climatological normal value for the period. Moreover, in summer, RGV was under the influence of ASAS, with lower rainfall index, which may have affected the dispersal of concentrations of pollutants emitted, which might have contributed to increase PM2.5 concentration. On other days, RGV was under the influence of the South Atlantic Convergence Zone (SACZ). In the winter, on the other hand, August, usually quite dry, was anomalously rainier in 2013. This may explain the higher concentration levels found in summer if we consider that emissions remained constant.

Although the WHO presents guidelines for air quality associated with PM_2.5_ (25 µg/m^3^ for short-term exposure, that is, average concentration of 24 hours), this same entity indicates that it is not possible to establish a minimum concentration of PM, below which no adverse health effects would occur. This study indicates that, even if the mean concentration of 24 hours of PM_2.5_ do not exceeded the upper limit of safety established by the WHO, even so one can identify the association between concentration of PM_2.5_ and incidence of acute respiratory events in children needing hospital care for a robust statistical model.

The study presents limitations because it was carried out in the short term, for only a period of winter and summer and with measurement of the pollutant on alternate days, given the unavailability of apparatus for continuous direct sampling of PM_2.5_. However, the use of portable devices enabled: sampling in places without automatic monitoring network of air quality, without electric power network and the conservation of samples for future analysis.

The results point to a significant relationship between concentration of PM_2.5_ and number of hospital care in children below 12 years, even with levels below the standards recommended by the WHO.

The complexity and variety of the sources of PM in RGV, besides increasing vehicular fleet, makes necessary to evaluate the concentration of PM_2.5_, its chemical composition, and the relationship of these variables with respiratory diseases. Further studies are needed to evaluate this relationship, especially in children, because they have more limited metabolic rates regarding exposure to chemicals.

## Footnotes

a Environmental Protection Agency (US). Model quality assurance project plan for the PM ambient air 2.5 monitoring program at State and Local Air Monitoring Stations (SLAMS). Durhan: Environmental Protection Agency; 1998 [cited 2014 Apr 8]. (Draft Report – EPA-454/R-98-005). Available from: https://www3.epa.gov/ttn/amtic/files/ambient/pm25/qa/totdoc.pdf

b Associação Brasileira de Normas Técnicas. MB-3422. Agentes químicos no ar - Coleta de aerodispersóide por filtração. Rio de Janeiro: Associação Brasileira de Normas Técnicas; 1991. ABNT (Associação Brasileira de Normas Técnicas) - MB-3422. Agentes químicos no ar - Coleta de aerodispersóide por filtração; 1991.

c Code of Federal Regulations. 40 CRF part 50, appendix L to part 50. Reference method for the determination of fine particulate matter as PM2.5 in the atmosphere. Washington, DC: Code of Federal Regulations; 2010 [cited 2014 Jul 10]. CFR (Code of Federal Regulations). 40 CRF Part 50, Appendix L. 2010. Available from: http://www.gpoaccess.gov/cfr/index.html

d Rede de Meteorologia do Comando da Aeronáutica (REDEMET) [cited 2014 Apr 8]. Available from: http://www.redemet.aer.mil.br/consulta_msg/consulta_de_mensagem.php?ID_REDEMET=kokhcn80hjtjmpq61eoelio0v6
